# Supplementary material for: Activation of MyD88-Dependent TLR Signaling Modulates Immune Response of the Mouse Heart during Pasteurella multocida Infection
Source: Microorganisms. 2023 Feb 4;11(2):400. doi: 10.3390/microorganisms11020400 (PMC9967429; doi:10.3390/microorganisms11020400)
Supplement: Supplementary file 1 [file microorganisms-11-00400-s001.zip › microorganisms-2177210-supplementary.pdf]

Supplementary Material

1. Supplementary Table

Supplementary Table S1. Primers used for validation of RNA-seq data.

Gene name	Primer sequence (5′-3′)	Amplicon length (bp)
Nfkbia	F: GCCAGTGTAGCAGTCTTGAC<br>R: CAGGTAGCCGTGGATAGAGG	117
Junb	F: CAGCCTTTCTATCACGACGAC<br>R: GGTGGGTTTCAGGAGTTTGTAG	96
Gadd45g	F: TCTACGAGTCCGCCAAAGTC<br>R: CACAGCAGAACGCCTGAATC	132
Iigp1	F: CAAATGAAGCAGATGGCAAACC<br>R: TTGTTAGAGAGCAGGAAGATTGG	130
C3	F: ACTTCTTCATTGACCTGCGGC<br>R: CGAGGACTTGGGAGGGATTT	197
Myd88	F: AAGCAGCAGAACCAGGAGTC<br>R: GCAGTAGCAGATAAAGGCATCG	150
Tbk1	F: ATCAAGAAGGCACGCATCCA<br>R: GGCTCATTGCTTTTGTGGCA	186
Stat1	F: GCCTCTCATTGTCACCGAAGAAC<br>R: TGGCTGACGTTGGAGATCACCA	123
Il4ra	F: GCTTGAAGAAGAACTCTAGTGTT<br>R: GATGTGGACTTGGACTCATTC	109
Cd14	F: TCGCTCAATCTGTCTTTCA<br>R: CTATCCAGCCTGTTGTAACT	95
β-actin	F: CCTCTATGCCAACACAGT<br>R: TAGGAGCCAGAGCAGTAA	92

2. Supplementary Figure

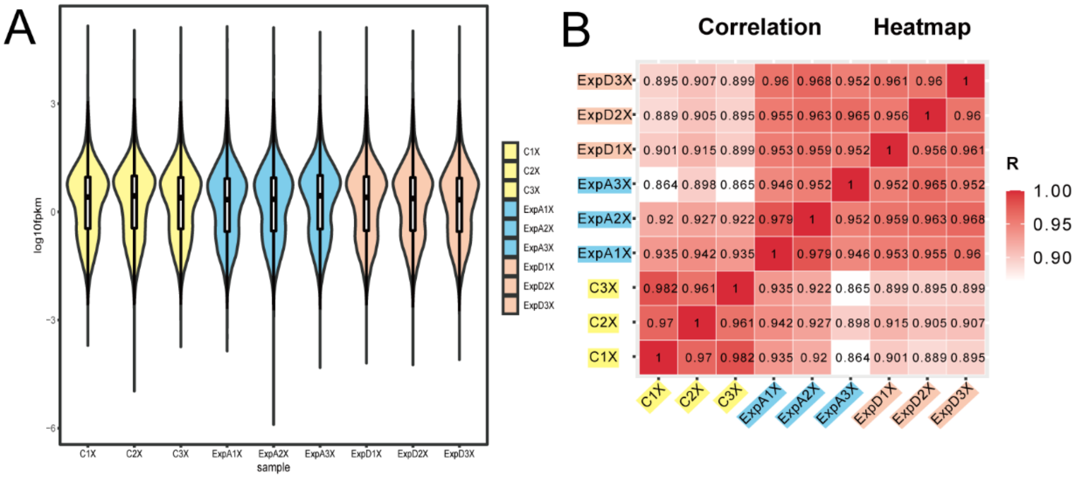

Supplementary Figure S1. The quality of sequencing data. (A) The abundance of gene transcript. (B) Correlation heatmap of samples. The gradient color barcode at the right indicates the minimum value in white and the maximum in red. If one sample is highly similar to another one, the correlation value between them is very close to 1.

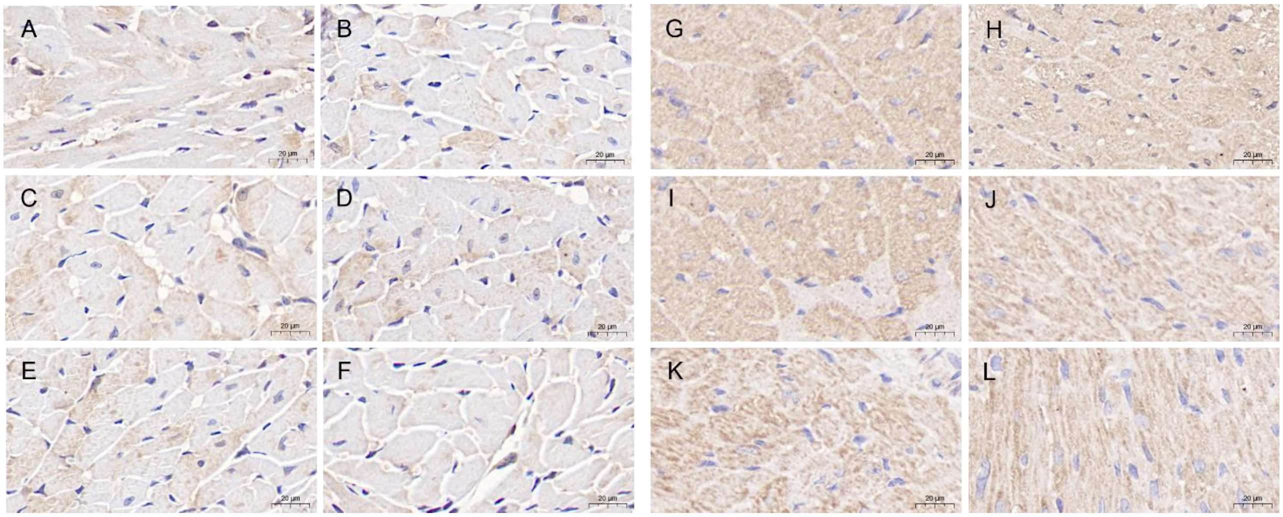

Supplementary Figure S2. The IHC of MYD88. (A-F) The control group, (G-L) the Pm HN02 group. The quantification of staining intensity was analyzed using these fields.
